# Adverse drug reaction profile of cisplatin-based chemotherapy regimen in a tertiary care hospital in India: An evaluative study

**DOI:** 10.4103/0253-7613.62412

**Published:** 2010-02

**Authors:** A. Surendiran, N. Balamurugan, K. Gunaseelan, Shahid Akhtar, K.S. Reddy, C. Adithan

**Affiliations:** Department of Pharmacology, JIPMER, Pondicherry, India; 1 Department of Pharmacology, JIPMER, Pondicherry, India; 2 Department of Radiotherapy, JIPMER, Pondicherry, India

**Keywords:** Chemotherapy, cisplatin, pharmacovigilance

## Abstract

**Aims:**

This prospective study was designed to monitor and analyze the pattern of occurrence of adverse drug reactions (ADRs) to cisplatin-based chemotherapy regimen in the cancer ward of a tertiary care hospital.

**Materials and Methods:**

Cancer patients who received cisplatin-based cancer chemotherapy were monitored for adverse reactions. The collected reports were analyzed for demographic and drug details, causality, preventability and severity of adverse effects. Causality was assessed by the World Health Organization (WHO) causality assessment scale and Naranjo's Algorithm. Preventability and severity of ADRs were assessed by modified Schumock and Thornton scale, modified Hartwig and Siegel scale respectively.

**Results:**

Among 51 patients, 48 developed ADRs to cisplatin chemotherapy. The reactions observed were nausea, alopecia, anorexia, vomiting, taste alteration, diarrhea, constipation, tinnitus, and hypocalcaemia. The WHO causality assessment scale indicated 69% “possible” and 31% “probable” but no “certain” reactions. Naranjo's Algorithm showed 62% “probable” and 38% “possible” reactions. Most of the reactions belonged to the category of “not preventable”. Reactions like nausea and vomiting belonged to the category of “definitely preventable”. Modified Hartwig and Siegel scale of severity assessment showed that most of the reactions were of “mild level 1” severity except for vomiting, diarrhea and hypocalcaemia, which were of “moderate level 3” severity.

**Conclusion:**

Cisplatin-based chemotherapy has a high potential to cause adverse effects. Most of the reactions were of milder nature but not preventable. The common adverse effects such as nausea and vomiting were preventable, but reactions like hypersensitivity reactions and anaphylaxis were not predictable.

## Introduction

Pharmacovigilance deals with detection, assessment and prevention of adverse drug reactions (ADRs).[1] Drug toxicity is a major limitation in providing healthcare to patients at a global level. It affects the patient's recovery as well the economy of healthcare. With the increase in production of various pharmaceutical products, newer drugs are being introduced every year. Hence, the need for an active surveillance system to remove the harmful drugs that have entered the market was well realized by the World Health Organization (WHO). This has been the basis for starting the International Drug Monitoring Program by the WHO.

The National Pharmacovigilance Program in India was started with the objectives of monitoring the safety of drugs and creation of an adverse drug reaction database for the Indian population.[1] The major aims of pharmacovigilance are early detection of unknown adverse reactions, detection of increase in frequency of known adverse reactions, identification of risk factors and dissemination of information.[2]

Cancer chemotherapeutic drugs like cisplatin have a very high potential for drug toxicity.[3] However, the number of ADR reports from the cancer wards to the pharmacovigilance center of our hospital was minimal. The reason for this paradox was not clear. It could be either due to gross underreporting of adverse drug reactions or due to effective preventive measures being adopted for the patients receiving cancer chemotherapy. As cisplatin is one of the most commonly used drugs for cancer chemotherapy, we did a focused monitoring of adverse drug reaction profile of cisplatin-based chemotherapeutic regimen. This study was designed to prospectively monitor and analyze the pattern of occurrence of ADRs to cisplatin-based chemotherapy regimen in the cancer ward of our tertiary care hospital.

## Materials and Methods

This prospective study was carried out by the Pharmacovigilance Center of a tertiary care hospital, among inpatients of oncology ward in the Regional Cancer Center of the hospital, over a period of two months. The Institutional Ethics Committee approval was obtained prior to initiation of study. The pharmacovigilance system consisted of notification forms, drop boxes[4] and a coordinated Drug Information Center. The cancer wards were fitted with drop boxes with a label for pharmacovigilance system along with notification forms by their side. The physicians had been instructed to fill the notification forms about the adverse drug reaction and put them in the drop boxes, which were then collected by the Pharmacovigilance Center. The ADR were assessed for causality, severity and preventability.

Cancer patients belonging to either gender and of all ages, who were receiving cisplatin-based cancer chemotherapy under any standard regimen, were included for the study. Those patients who did not receive cisplatin as part of the drug regimen were excluded from the study. The patients received cancer chemotherapy as per the assessment of the treating physician. No changes in the treatment decision, schedule or duration were made as a part of the study. The patients admitted for cancer chemotherapy and receiving cisplatin as part of the regimen were monitored for adverse effects till their discharge from hospital.

All the patients received pre-medication with intravenous ranitidine, dexamethasone and ondansetron to avoid emesis, as cisplatin is a highly emetogenic drug.[5] They were also administered post-medication with intravenous mannitol to avoid nephrotoxicity. Monitoring for adverse effects was done based on daily questioning for symptoms. The collected reports were documented in the case report form and analyzed for demographic details, drug details, causality, preventability and severity of adverse effects. Causality was assessed by both WHO causality assessment scale[6] and Naranjo's Algorithm.[7] Preventability was assessed by modified Schumock and Thornton scale.[8] The severity of ADRs was assessed by modified Hartwig and Siegel scale.[9]

The WHO causality assessment scale is recommended by the WHO Uppsala Monitoring Center, which is the WHO collaborating Center for International Drug Monitoring,[6] for evaluation of the causal relationship of drugs to its adverse effects. The Naranjo's Algorithm, a questionnaire designed by Naranjo *et al*. consists of objective questions with three types of responses - yes, no or do not know. Scores are given accordingly and the drug reaction can be classified as definite, probable or possible. The modified Schumock and Thornton scale classifies ADRs as definitely preventable, probably preventable and not preventable based on a set of questions for each level. The modified Hartwig and Siegel scale classifies severity of ADR as mild, moderate or severe with various levels according to factors like requirement for change in treatment, duration of hospital stay, and the disability produced by the adverse drug reaction.

No invasive investigation was undertaken or suggested to the treating physician by the investigator as a part of the study. The drug effects which were described by the patients and effects which were diagnosed and reported by the physician were documented.

## Results

The adverse drug reaction reports were obtained by regular questioning of patients by the investigator in the evening hours of working days. Out of 48 patients with adverse drug reactions, only one case was spontaneously reported to the Regional Pharmacovigilance Center by the health care worker in the ward. At the end of the study period, a total of 51 patients who received cisplatin-based chemotherapy were monitored for adverse reactions till their discharge from hospital. This included 17 male patients and 34 female patients. The various indications for its use were carcinoma of cervix, lung with metastasis, oral cavity, ovary and thymomas. The most common indication was cervical carcinoma. Among the 51 patients observed, 48 developed adverse reactions to the chemotherapy regimen. This included 25 patients receiving cisplatin alone, 22 patients receiving cisplatin along with one additional anti cancer agent (5-fluorouracil, paclitaxel, ifosfamide, gemcitabine) and one patient receiving cisplatin with two additional anticancer agents (adriamycin and ifosfamide). Twenty patients who developed ADRs received concomitant radiotherapy. Only three out of 51 patients did not develop ADRs to cisplatin chemotherapy.

**Figure 1:**
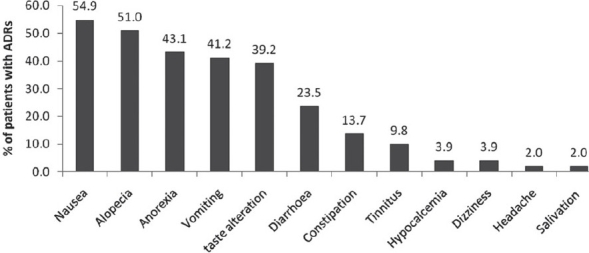
Frequency of adverse drug reactions to cisplatin chemotherapy

The ADRs observed in the patients were nausea, alopecia, anorexia, vomiting, taste alteration, diarrhea, constipation, tinnitus, and hypocalcaemia [Figure 1]. Assessment of causality by WHO causality assessment scale[6] indicated that 69% of the reactions belong to the category “possible”, followed by the category “probable” with frequency of 31% [Table 1]. There were no “certain” reactions as re challenge was not attempted in any of the patients. However, the grade of causality for each ADR remained low due to presence of co-administered drugs. As per Naranjo's Algorithm[7] almost 62% of the ADRs were categorized as “probable” with score ranging from 5 - 8 and 38% of the ADRs were categorized as “possible” with score ranging from 1 - 4 [Table 2]. Assessment of preventability of the ADR was done based on modified Schumock and Thornton scale[8] [Table 3]. Most of the ADRs belonged to the category of “not preventable”. However, the more common reactions like nausea and vomiting belonged to the category of “definitely preventable”. Based on modified Hartwig and Siegel scale[9] of severity assessment, most of the reactions were of less severity categorized as “mild level 1” severity, except for vomiting, diarrhea and hypocalcaemia, which were categorized as “moderate level 3” severity.

**Table 1:** Causality assessment of individual adverse drug reaction by WHO Causality Assessment Scale.

*ADRs*	*Number of ADRs*				*Total*
		
	*Certain*	*Probable*	*Possible*	*Unlikely*	
Nausea	-	7	21	-	28
Vomiting	-	3	18	-	21
Anorexia	-	-	22	-	22
Diarrhea	-	2	10	-	12
Tinnitus	-	1	4	-	5
Taste alteration	-	13	7	-	20
Alopecia	-	16	10	-	26
Hypocalcaemia	-	2	-	-	2
Constipation	-	-	7	-	7
Total (%)	-	44 (31%)	99 (69%)	-	143

**Table 2:** Causality assessment of individual adverse drug reaction by Naranjo's Algorithm.

*ADRs*	*Number of ADRs*			Total
		
	Possible*	Probable*	Definite*	
Nausea	11	17	-	28
Vomiting	11	10	-	21
Anorexia	22	-	-	22
Diarrhea	7	5	-	12
Tinnitus	4	1	-	5
Taste alteration	-	20	-	20
Alopecia	-	26	-	26
Hypocalcaemia	-	2	-	2
Constipation	-	7	-	7
Total (%)	55 (38%)	88 (62%)	-	143

* Possible (Total Score 1-4), Probable (Total Score 5-8), Definite (Total Score >9)

**Table 3:** Assessment of preventability by Modified Schumock and Thornton Scale

*Definitely preventable*	*Probably preventable*	*Not preventable*
Nausea	Diarrhea	Anorexia
Vomiting		Alopecia
		Constipation
		Hypocalcaemia
		Taste alteration
		Tinnitus

## Discussion

Cisplatin is a commonly used anti neoplastic agent. Some of the well documented ADRs of this drug include nausea, vomiting[5,10] renal toxicity,[11–13] ototoxicity,[14] peripheral neuropathy,[15] hypersensitivity reactions[16] and electrolyte disturbances.[17–22] Most of the ADRs documented in this study comprised one or more of these reactions. Although adequate pre-medication with parenteral dexamethasone, ranitidine and ondansetron were given to each patient, the frequency of nausea and vomiting remained high due to the high emetogenic potential of cisplatin. The most frequent adverse effects include nausea, alopecia, anorexia and vomiting [Figure 1]. The study has demonstrated the need to improve the management of nausea and vomiting, since the rates of prevention of these expected adverse effects of cisplatin were poor. Some of the rarer reactions include hypocalcaemia, headache, salivation and dizziness. Pretreatment values of complete hemogram were taken for every patient before each cycle and post treatment assessment was done only if clinically indicated. There were no reports of hematological disturbances.

Nausea and vomiting are very common side effects of cancer chemotherapeutic drugs.[10] These drugs may induce vomiting by both a central action on the chemoreceptor trigger zone (CTZ) and a peripheral action on the gastrointestinal tract. The dominant receptors in the CTZ located in the floor of the fourth ventricle are serotonin Type 3 (5-HT3) and dopamine Type 2 (D2).[23] As serotonin receptors in the brain are involved in the mechanism of acute onset vomiting, ondansetron has a definite role in its prevention.[24] Ototoxicity can be more severe in children. It can manifest as tinnitus and loss of hearing in the high frequency range.[14] Electrolyte disturbances such as hypomagnesaemia, hyponatremia, hypocalcaemia and hypokalemia are known to occur.[17–22] We observed that 51% of patients studied developed alopecia. Earlier studies have shown conflicting results implicating cisplatin as a common causative agent for alopecia as well as an unlikely agent for alopecia.[25–27] In our study, out of 26 patients who developed alopecia, 14 were on monotherapy with cisplatin, seven had concurrent ifosfamide, two had concurrent 5-fluorouracil, and one each had paclitaxel, adriamycin plus ifosfamide and gemcitabine. Among patients who developed alopecia, more than 50% were on monotherapy with cisplatin. Diarrhea can occur due to mucosal cell toxicity. Animal studies have demonstrated the effect of cisplatin causing specific mitochondrial oxidative DNA damage in gastro intestinal mucosal cells and increased gastro intestinal permeability, an indicator of toxicity.[28]

A study by Bahl *et al*,[26] on 40 patients with locally advanced non small cell lung cancer, treated with cisplatin and etoposide, described the ADR pattern to cisplatin. However, our study included all patients who were under therapy with cisplatin-based chemotherapy regimen irrespective of their diagnosis. The frequency of alopecia was reported as 88% in their study compared to 51% in our study. Occurrence of nausea and vomiting had similar frequency in both studies. Anorexia had a slightly higher frequency in them (52%) compared to our study (43%). Though these studies reported hematological abnormalities like leucopenia, anemia and thrombocytopenia, we did not observe such effects.

In the present study, almost all the ADRs except anorexia and hypocalcaemia assessed as “possible” with lower level of causality by WHO scale had been identified as “probable” with higher level of causality based on Naranjo's Algorithm. This shows the more objective nature of Naranjo's Algorithm. There were no “certain” drug reactions as the patients were not subjected to re challenge of the drug. There were no “unlikely” drug reactions as the investigator was trained in methods of pharmacovigilance and such complaints were avoided.

Most of the ADRs were not preventable due to the poor predictability of the ADRs and poorly understood mechanisms to explain their cause. However, with adequate pre-medication, common ADRs like nausea and vomiting can be effectively controlled. This brings out the possible toxicity that the treating physician should anticipate and counsel the patient adequately prior to starting of therapy.

Most of the reactions were of less severity and there would be no strong indication to change or withhold the drug for milder adverse effects. Similar studies can be used as audit tools for iatrogenic adverse events and assessment of prevention of expected adverse drug reactions.

There is a need for effective pharmacovigilance in India owing to absence of Indian data on adverse effects and the genetic diversity of the Indian population.[29] This study had carried out a focused ADR monitoring on cisplatin-based chemotherapy for cancer patients, though there were anecdotal case reports of adverse reactions to cisplatin.

In conclusion, cisplatin-based chemotherapy has a high potential to cause various adverse effects in cancer patients. Most of the adverse drug reactions in this study were mild, but not preventable; hence they do not affect the treatment. It should also be noted that though the common adverse effects such as nausea and vomiting were preventable, most of the idiosyncratic reactions like hypersensitivity reactions and anaphylaxis were non-specific and not predictable. This study also emphasizes the need to improve pharmacovigilance awareness among physicians in order to improve the pharmacovigilance system in India.
